# A Multi-Host Agent-Based Model for a Zoonotic, Vector-Borne Disease. A Case Study on Trypanosomiasis in Eastern Province, Zambia

**DOI:** 10.1371/journal.pntd.0005252

**Published:** 2016-12-27

**Authors:** Simon Alderton, Ewan T. Macleod, Neil E. Anderson, Kathrin Schaten, Joanna Kuleszo, Martin Simuunza, Susan C. Welburn, Peter M. Atkinson

**Affiliations:** 1 Institute of Complex System Simulation, School of Electronics and Computer Science, University of Southampton, Southampton, United Kingdom; 2 Geography and Environment, Faculty of Social and Human Sciences, University of Southampton, Southampton, United Kingdom; 3 Lancaster Environment Centre, Lancaster University, Lancaster, United Kingdom; 4 Division of Infection and Pathway Medicine, Edinburgh Medical School – Biomedical Sciences, College of Medicine and Veterinary Medicine, The University of Edinburgh, Edinburgh, United Kingdom; 5 The Royal (Dick) School of Veterinary Studies and the Roslin Institute, University of Edinburgh, Roslin, United Kingdom; 6 Department of Disease Control, School of Veterinary Medicine, University of Zambia, Lusaka, Zambia; 7 Faculty of Science and Technology, Engineering Building, Lancaster University, Lancaster, United Kingdom; 8 School of Geography, Archaeology and Palaeoecology, Queen’s University Belfast, Belfast, United Kingdom; Johns Hopkins Bloomberg School of Public Health, UNITED STATES

## Abstract

**Background:**

This paper presents a new agent-based model (ABM) for investigating *T. b. rhodesiense* human African trypanosomiasis (rHAT) disease dynamics, produced to aid a greater understanding of disease transmission, and essential for development of appropriate mitigation strategies.

**Methods:**

The ABM was developed to model rHAT incidence at a fine spatial scale along a 75 km transect in the Luangwa Valley, Zambia. The method offers a complementary approach to traditional compartmentalised modelling techniques, permitting incorporation of fine scale demographic data such as ethnicity, age and gender into the simulation.

**Results:**

Through identification of possible spatial, demographic and behavioural characteristics which may have differing implications for rHAT risk in the region, the ABM produced output that could not be readily generated by other techniques. On average there were 1.99 (S.E. 0.245) human infections and 1.83 (S.E. 0.183) cattle infections per 6 month period. The model output identified that the approximate incidence rate (per 1000 person-years) was lower amongst cattle owning households (0.079, S.E. 0.017), than those without cattle (0.134, S.E. 0.017). Immigrant tribes (e.g. Bemba I.R. = 0.353, S.E.0.155) and school-age children (e.g. 5–10 year old I.R. = 0.239, S.E. 0.041) were the most at-risk for acquiring infection. These findings have the potential to aid the targeting of future mitigation strategies.

**Conclusion:**

ABMs provide an alternative way of thinking about HAT and NTDs more generally, offering a solution to the investigation of local-scale questions, and which generate results that can be easily disseminated to those affected. The ABM can be used as a tool for scenario testing at an appropriate spatial scale to allow the design of logistically feasible mitigation strategies suggested by model output. This is of particular importance where resources are limited and management strategies are often pushed to the local scale.

## Author Summary

African trypanosomiasis is a parasitic disease which affects humans and other animals in 36 sub-Saharan African countries. The disease is transmitted by the tsetse fly, and the human form of the diseases is known as sleeping sickness. Infectious disease transmission has traditionally been modelled using techniques that consider the impact on a population as a whole (e.g. compartmentalised models such as SIR). For diseases such as sleeping sickness, which are often prevalent in sparsely populated rural environments, these models don’t always capture the spatial and demographic heterogeneity within an area, and the varying exposure to the disease that this can cause. This research introduces a novel agent-based model to the field, which incorporates spatial data for the Luangwa Valley case study, along with demographic data for its inhabitants. Tsetse and potential human and animal hosts are modelled at the individual level, allowing each contact and infection to be recorded through time. Through modelling at a fine-scale, we can incorporate detailed mechanisms for tsetse birth, feeding, reproduction and death, while considering what demographics, and which locations, have a heightened risk of disease.

## Introduction

Human African trypanosomiasis (HAT), also known as sleeping sickness, is a parasitic disease which poses a significant disease burden in affected communities living in HAT foci across sub-Saharan Africa [1, 2]. HAT is caused by two sub-species of the protozoan parasite *Trypanosoma brucei* s.l.: *T. b. rhodesiense*, in eastern and southern Africa and *T. b. gambiense* in West Africa [3]. The parasite is transmitted cyclically by tsetse flies (genus: *Glossina*), in which it undergoes a complex life-cycle [4]. *T. b. rhodesiense* HAT (rHAT) is a zoonoses, affecting a wide range of wildlife [5, 6] and domestic animals, particularly cattle [7], presenting in humans as an acute disease [8].

HAT epidemics display characteristic periodicity [9, 10]; cases are currently declining across sub-Saharan Africa, in part attributed to improved approaches to case finding and vector control [11]. HAT co-exists in natural ecosystems with a suite of trypanosomes that affect the health of domestic livestock. African animal trypanosomiasis (AAT) was described in 1963 as being “one of the most important factors restricting economic development in Africa today” due to widespread disease, and overstocking of cattle in tsetse-free areas [12]. A greater understanding of disease transmission in natural and changing ecologies will aid HAT and AAT strategies for disease prevention and control, improving the health and wellbeing of humans, livestock and wildlife. Mathematical modelling of neglected tropical disease (NTD) transmission systems can have a significant impact on intervention strategies and feed into policy formulation [13]. Whether through assessing theoretical interventions [14], investigating vector mortality (e.g. [15]), or modelling vector-host interactions (e.g. [16–18]), transmissible disease modelling can be undertaken using a range of methods, at a range of scales, targeted at various aspects of transmission and control. For HAT, multiple approaches have been applied including mathematical modelling of antigenic variation in trypanosomes [19], investigation of local scale migrations [20], and examining the implications of activity-related movements through agent-based models (ABM) [17]. Traditional compartmentalised modelling approaches have also been applied to HAT. Using a ‘host—vector model’ [21], the impact of a vector control strategy for *T. b. gambiense* HAT (gHAT) in the Niari focus, Central Africa was modelled, and suggested that a 50% reduction in vector density could prevent a gHAT outbreak [22].

Compartmentalised models have been criticised for their inability to represent interdependent processes such as how individuals interact with each other and their environment through space and time [23]. Where multiple parameters are at play as for vector-borne diseases with multiple hosts, susceptible-infected-susceptible (SIS) models may not fully capture overall disease circulation within that environment [24].

This paper describes the development of an ABM for rHAT/AAT from data derived from a detailed rHAT, AAT, and tsetse ecological survey, undertaken in 2013, in Eastern Province, Zambia, combined with published data and information from experts in the field. The ABM was applied to answer the following research questions; Who is at greatest risk? Where is infection most likely to occur? Where are cases likely to reside? And to whom and where should control strategies be targeted? Specifically the ABM can be used to identify activities that generate large amounts of simulated infection in the human population, and these results can be explored, in depth, to identify emergent demographic and spatial patterns. For example: does sparse provision of schools in an area generate the need to travel long distances to access education which heightens exposure? And, in the absence of a borehole, do frequent trips to the river increase connectivity between vector and host? The results are meaningful in relation to the situation at the study site, but the case study also serves as an exemplar of what is possible with an ABM in the vector-borne disease context.

Agent-based modelling enables the incorporation of fine-scale spatial and demographic information. Since exposure is key to HAT infection, agent-based modelling of human movements that expose individuals to hazards that vary through time and space has implications for risk-mitigation that can feed into policy [25]. Agent-based modelling has been previously applied to model gHAT in Cameroon, but the ABM did not incorporate realistic geographical data, linked to a geographical information system (GIS) [17].

ABMs model disease transmission using a completely spatialized approach, incorporating factors often overlooked (e.g. human behaviour and activity-based movement, density and mobility of vectors and contribution of additional hosts). Agent-based modelling can be applied to acquire preliminary knowledge of disease systems, including patterns of interactions between individuals within the network [17]. An agent-based modelling representation of the dynamics of people-vector contacts in space and time can facilitate investigation of scenarios that have not been observed or previously explored. For rHAT the modelling of people-vector contacts is of particular significance given that mitigation strategies focus on the control of tsetse fly movement and density. The benefits of incorporating geographical data into epidemiological models is comprehensively described in the literature, particularly as landscape features largely control the connectivity between hosts and vector habitats, inhibiting movement, and ultimately modifying disease risk ([26, 27], e.g.).

One of the least well integrated factors in traditional landscape epidemiology is human behaviour [27]. Different perceptions of risk between sexes and by permanent and part-time residents within endemic areas, influences the adoption of preventative measures and ultimately varies transmission risk [28]. The modelling of social contact structure, together with daily activity routines, are key when attempting to model the diffusion of infectious diseases, and for design of policies for disease mitigation ([29, 30], e.g.). Agent-based modelling can capture the stochastic nature of human agent’s infection [31], with the model landscape creating variation in the timing, location and probability of infection as a result of its influence on variability in contact patterns [32, 33].

## Materials and Methods

### Ethics Statement

The field study described in the following sections gained ethical approval from ERES Converge, a Zambian private research ethics board.

### Study Area

The Luangwa Valley, in Zambia is an extension of the Great Rift Valley in East Africa, traversing Eastern, Northern and Muchinga Provinces. Mid-Luangwa Valley has recently experienced increased immigration of people seeking fertile land. Land pressure has resulted in human settlement in tsetse-infested areas, previously avoided, for fear of disease risk to introduced livestock. Households grow cotton as a cash crop and maize and groundnuts for home consumption [34].

HAT is endemic in the Luangwa Valley, first being observed in 1908 [35]. *G. m. morsitans* was not originally considered a vector of HAT in the valley, despite 50% of domestic and game animals in the valley having been observed to harbour trypanosomes [36]. HAT infection was assumed to have originated from Dowa, Malawi, where *G. p. palpalis* were reported. In the early 1970s, a large HAT outbreak occurred in Isoka (241 case in 3 years) attributed to fly encroachment from Luangwa [37]. Game had been observed to reside in Isoka for several months during the rainy season, migrating away during the dry season. Buyst [37] speculated that during the dry season, starved tsetse took feeds from humans in villages and that a predominance of infections in women and children (four times that in men) was due to them remaining close to the village throughout the year (even though women and children did venture into tsetse habitat to fetch firewood and water). Men were considered to be least at risk as they ventured further afield to hunt. In 1973, early diagnosis and improved treatment methods were introduced, and case numbers fell [38]. Today, cases of rHAT continue to be reported in the Luangwa Valley.

The area in which this study was undertaken is an area undergoing substantial transition; with constant immigration and land pressure, new immigrants to the valley are forced to occupy increasingly marginal land where they at greater risk to exposure to rHAT and their animals from AAT [34]. Risk factors include human proximity to the large wildlife reservoir in the South Luangwa National Park to the north-west [5], and ever-increasing livestock (and human) density on the plateau. Little is known concerning tsetse-trypanosome-human interaction in the region and the ABM can enable exploration of the risk within these communities.

#### Study transect

**Fig 1:**
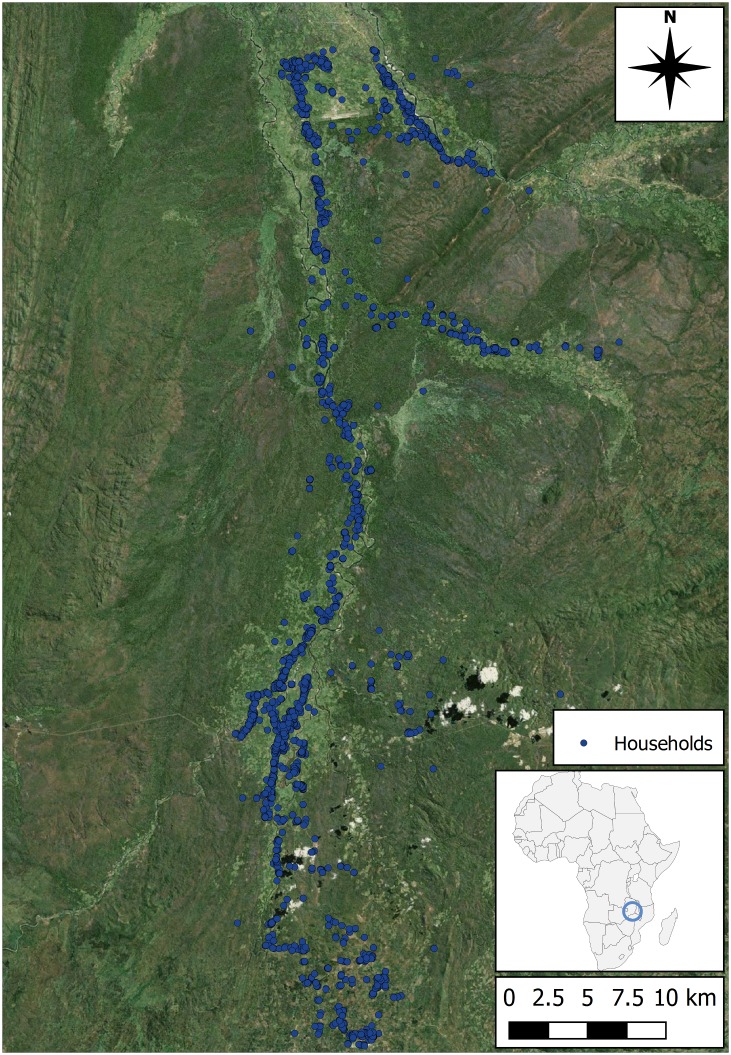
Map of the study area size and location. Households from the census included in this modelling study are indicated as blue circle (produced using Landsat 7 imagery available from USGS).

The study area spans a sparsely populated region of the rural Eastern Province of Zambia (Fig 1). Villages are small (between 5 and 20 households, S1 Fig) and inhabitants are predominantly subsistence farmers. The data collection area consists of a 75 km transect which starts close to Mfuwe airport in the north, and runs southwards along the Lupande River and its tributaries.

Three primary sources of data collection were used to generate an ABM of the study area: tsetse fly distribution data to highlight the risk areas for disease transmission, a sample of demographic specific routines used by inhabitants across the study area to gain an indication of the prominent activity patterns, and census data for humans, cattle and other domestic animals in the region so that the whole population of the study area could be modelled based on an accurate spatial distribution. Data describing human routines and tsetse fly distribution were acquired using human movement questionnaires and tsetse fly rounds, respectively, together with human and animal censuses in the region. Fieldwork was undertaken periodically from 2012–2014 as a part of the Dynamic Drivers of Disease in Africa Consortium (DDDAC) research programme. The following sections describe the collection and processing of sensor data (to identify routes between villages and resources), before presenting tsetse fly round data and the techniques used to process it for incorporation into the ABM. Finally, the collection and processing of animal and human census data are described, along with information about human daily routines.

### Sensor Data and A* Pathfinding

To identify fine scale features and produce plausible paths between villages and resources, satellite sensor and fine-scale aerial imagery were required. Aerial imagery with a spatial resolution of 11 m was used to digitise fine scale features (e.g. narrow roads and river sections that might not be identifiable at the 30 m satellite sensor image resolution). The locations of known resources (e.g. schools, markets and boreholes) were digitised at this scale using GPS coordinates from fieldwork and census data, together with village locations, to provide home and target locations for pathfinding input. A land classification layer was produced initially using 30 m spatial resolution Landsat 7 satellite sensor imagery, classifying bare-land, crops, bush/forest and built environment. This classification was subsequently converted to an 11 m spatial resolution, so that fine scale features could be added to the classification, such as roads and river.

Plausible routes between each village and each resource type were calculated using this land classification image as an input for an A* pathfinding algorithm, described in [39]. The A* pathfinding technique provides a logical balance between straight line movement from home to destination, and absolute least cost path. The pathfinding technique for paths to river and borehole resources for water collection, was calibrated comparing simulated path choices and walk times to those provided by local inhabitants from questionnaire data (calibration data provided as supplementary information in [39]). The same technique was applied to markets, schools, crop areas and firewood locations in this study. An example product of the A* pathfinding algorithm and land classification data is shown in S2 Fig, highlighting navigation around physical features between source and destination.

### Tsetse Data Collection and Processing

**Fig 2:**
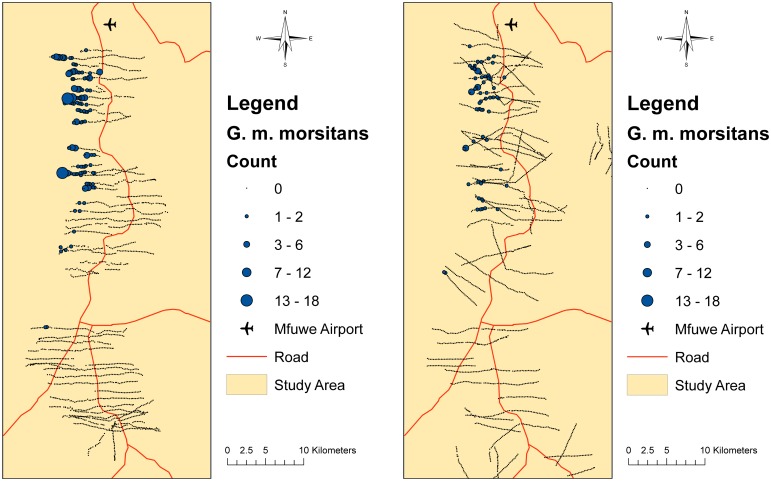
Transect locations of tsetse fly data collection carried out in June (left) and November (right) 2013. Proportional circles highlight tsetse density and distribution.

Two intensive tsetse surveys were undertaken in June and November 2013, using black screen fly-round transects, and Epsilon traps (see Fig 2). Although, the model presented in this paper does not incorporate the impact of seasonality on the tsetse population, focusing rather on a “typical day”, it should be noted that the dry, hot period (late August to December) can cause the tsetse population to reduce in distribution and density and the migration of wildlife away to water sources, whereas the rains can be both beneficial (increased vegetation and resting places) and detrimental (the washing away of pupae).

To derive an estimate of the total number of tsetse in the region, the area of the whole tsetse study was calculated, by summing the total area covered by each individual fly round transect, whether tsetse were caught or not. The study area was 15 times larger than the transect area and it was assumed that for every tsetse caught 14 were missed that such as to estimate a total tsetse population of 5,250 flies.

**Fig 3:**
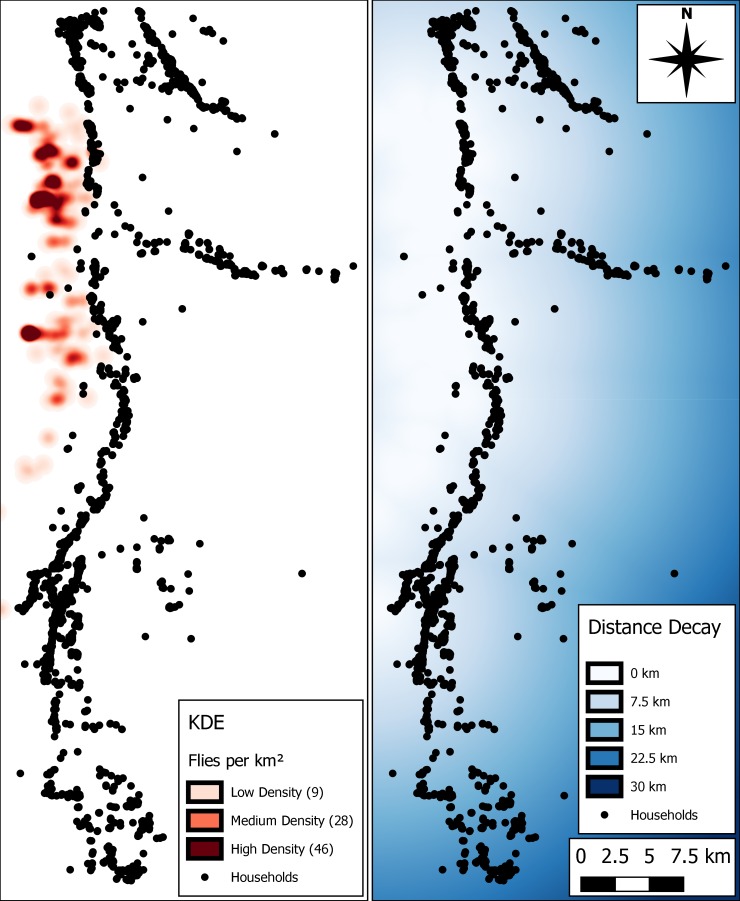
Estimated distribution of the tsetse fly population in the study area. Left: heat map of the kernel density estimate (KDE), Right: fly movement inhibitor, where colour gradient represents distance decay from non-zero tsetse density estimates based on the KDE.

To estimate tsetse density and distribution from the transect data sample, a kernel density technique was used to generalise point data. Assuming an individual fly moves within an 800 m radius of its initial location in a day [40], the point data acquired in the transect study on presence of tsetse actually represents a greater areal influence within the study region. To estimate areal influence, a kernel density estimate (KDE) was produced, generalising to account for movement patterns and absences, reasoning that each caught fly could have started the day 800 m away from the catch site in any direction. The KDE heat map output is shown in Fig 3 (left) with higher densities of tsetse distributed in the north-west of the study area closer to the South Luangwa National Park, with small, but non-zero values stretching further south. The image on the right, illustrates an area of all non-zero KDE values (dark blue), with distance decay away from this region. The non-zero KDE area is referred to as the ‘tsetse-fly zone’.

### Census Data and Human Movement Routines

A human and animal census of the study area produced demographic data for 16,024 human inhabitants, 2,925 cattle, and 11,576 other domestic animals (goats, pigs, donkeys, cats and dogs). Census data included age, gender, tribe and cattle ownership throughout the study region, together with number and distribution of cattle and other domestic animals per household. Information was processed to determine human and animal agent distributions across the villages previously digitised in the study area. A sample of 94 households provided responses to a questionnaire on human movement that determined the frequency and times of day that each respondent travelled to farm, school, market, water and firewood resources, together with trips to tend to cattle (if applicable). Responses were organised by age and gender, generating a set of possible daily routines that might be undertaken, for example, by a female of between 18 and 50 years. Further information on collection of routine data can be found in the supplementary material (S1 File).

### Methods

#### ABM framework

Pre-processing provided all the data required to initialise and run the ABM, including land classification of the study area, location of households and resources, human and animal populations living in these villages, a sample of resource-seeking routines sorted by gender and age, and a set of plausible paths from each village to undertake these routines. For tsetse, an estimate of the total apparent population size, density and distribution was provided. Four agent types were included in the ABM, together with an areal representation of wildlife. Human, cattle, other domestic animals and tsetse used in the ABM were constructed as four separate classes, with populations modelled 1:1 with the data collected in the census (e.g. 16,024 human agents) and the estimated tsetse population discussed previously. Each class had its own initial information and storage structures for events that occurred through the simulation. The ABM was written in Python 2.7 using an object-oriented framework, and run on the Lancaster University High End Computing (HEC) Cluster, with all spatial data being processed using Quantum GIS 1.8.0.

#### Human agents

Human agents are initialised using the demographic census data and a daily routine based on the questionnaire data. Each human agent is given their home coordinates, village number, village name, house number (in the village), age and gender. If the village and household numbers corresponded to a location where cattle were found in the animal census, cattle agents are allocated to this household, and a human agent (aged 15–60 years old) is attributed the responsibility for looking after those cattle. Cattle agents have the same information as human agents, with the addition of a specific owner agent ID. Routines are represented by a list of trip frequencies to the resources associated with the human agents including farming, water collection, attending school, going to market and collecting firewood. Information is coupled with the time period under which these trips are undertaken (morning, lunchtime, afternoon, evening). A small proportion of routines include activity relating to cattle care (watering and grazing). A human agent attributed with looking after cattle, is assigned randomly a routine corresponding to their age and sex that included cattle management. Each human agent’s village is allocated a set of pre-defined (i.e. A* generated) paths to local resources. In the absence of data concerning children aged 0–5, children of this age stay within the village throughout the course of the simulation.

#### Cattle and other domestic animal agents

Cattle were simulated using a ‘cattle agent’ class and assigned an owner and village location at initialisation. Each cattle agent carries out cattle associated activities with its owner (grazing and cattle watering), while remaining in the village to undertake other human tasks. Other domestic animals do not leave their village during the simulation, moving randomly within a 50 m range of the village, being included largely as a buffer for tsetse biting.

#### Tsetse fly agents

Parameter values attributed to each agent within the tsetse class at the initiation of the main simulation are shown in Table 1. There are 5,250 tsetse agents at the start of the simulation based on the estimate of tsetse population size. Fly agents are described as teneral (newly emerged and unfed) or non-teneral.

**Table 1:** Parameter values for tsetse fly agents present at ABM initialisation. N.B. Anything with a range is assigned randomly within that range.

Parameter	Value	Source
**Age**	All flies are mature (non-teneral) of age: Male: 1–40 days; Female: 1–100 day	Estimated range based on field and laboratory variation in G. morsitans lifespan and mortality, e.g. [4, 41]
**Gender**	Male (33%), Female (66%)	[42, 43]
**A priori mating**	All female tsetse aged 2 days or over have mated	[44]
**Disease susceptibility**	16% chance of tsetse fly being susceptible to the infection	[45]
**Infected midgut—immature infection**	0.1% of the total tsetse population starts with a midgut infection (taken from the susceptible population).	PCR on tsetse indicated 2.2% *T.b. brucei* prevalence (Martin Simuunza, pers com). Reduced to 0.1% to account for the inability of PCR to distinguish between refractory flies possessing parasite DNA from feeding on an infected animal, and flies with a cyclical infection [46], from comparisons between PCR and dissection data [47].
**Infective salivary gland—mature cyclical infection**	In tsetse with a midgut infection, the probability of having a mature, salivary infection at simulation start is gender dependent: Male—21%; Female—4%	[48]

#### Representing wildlife

The influence of wildlife was considered differently to other possible hosts in the model due to uncertainties regarding their density and distribution, given the lack of empirical data available for the study area. Due to the proximity of the tsetse fly zone to the South Luangwa National Park, and the higher concentration of tsetse, it is likely to be a zone of higher wildlife density, particularly away from human settlement. This has been confirmed by sightings of different wildlife by local villagers from the fieldwork questionnaires. The area defined by this study as a tsetse fly zone, was also considered evenly distributed with wildlife. An additional buffer for tsetse biting was therefore generated, providing an additional source of infection for tsetse.

#### Infected hosts at the start of the simulation

The number of infected host agents at the start of the simulation is representative of data collected under DDDAC (Table 2). All infected agents are randomly chosen to be individuals who start in, or are adjacent to the tsetse fly zone (within 2 km buffer).

**Table 2:** Numbers of infected agents at ABM initialisation.

Agent Type	Estimated Population Infected with *T.b. rhodesiense*
Cattle	6
Goat	5
Dog	1
Pig	2
Human *	10

*Communication with medical teams in the region suggested 2 human cases of sleeping sickness in the past year. Due to the documented issues of under-reporting (e.g. 12 cases for each reported [49]), an estimated number of 10 human infections was used in this study.

N.B. The implications on average infection of different numbers of infected humans and cattle were explored as part of the sensitivity analysis. Results for the different scenarios can be found in S2 File.

The impact of infected wildlife was incorporated using data from a recent study of the wildlife reservoir in the Luangwa Valley in which *T. brucei* s.l. were detected in approximately 5% of all wildlife sampled [5]. Using an estimated ratio of 10:1 for *T.b. brucei* and *T.b. rhodesiense* infections in the region [50], a prevalence of 0.5% *T.b. rhodesiense* was attributed to wildlife in this study [5].

#### Space

Three spatial resolutions were applied within the model, with each resolution level providing different information to the agents. At the coarsest scale, 1 km ‘region squares’ are used to query the proximity of individual agents with other agents in the simulation. Given, for example, a tsetse fly agent sitting in one region square, all agents sitting outside of this region square, or its eight neighbours (Moore neighbourhood), are discounted when considering finer calculations of interaction due to their distance. This allowed compilation of ‘close’ host and tsetse agents for further investigation, and eliminates unnecessary fine scale calculations of distances to agents that are, for example, 30 km away. Should two agents be considered sufficiently close after this filtering, distances are calculated on a continuous scale to the nearest metre. Distances between tsetse and bush/forest resting sites are also considered on this continuous scale. Finally, agents can query land cover classification in the simulation at an 11 m spatial resolution; the resolution of the land classification input layer discussed previously. This layer is used for identification of close bush resting sites for tsetse agents, along with modifying human agent movement speeds along the pre-processed paths between home and resources.

#### Time

Time was split into 2,400 time-step (or tick) days, meaning that each tick represented 36 seconds of simulated time. The temporal resolution was very high to reduce the chance of missing any possible contacts between vectors and hosts. If this resolution was coarsened, it would be increasingly possible that interactions would be missed since movements are represented as small jumps every tick. As the tsetse move quickly, a longer period than 36 seconds might mean that a tsetse fly could ‘pass over’ a possible bite target between ticks, with the bite target being out of smell range before and after. The more frequent the tick, the smaller the jumps, and the less chance of missing potential interactions. A finer temporal resolution would have increased simulation length, and caused too great a demand for processor power and memory.

#### Model dynamics

A single day is described in the simulation from the point of view of a human agent and a tsetse fly agent, highlighting their individual behaviour.

#### Humans

A trip to a resource is selected at random for each human by time period (morning, lunchtime, afternoon, evening) based on an assigned routine. When a trip is completed successfully, it is removed from their daily routine (reset at the end of each day). If a trip cannot be completed by the end of the day, it is rolled over into the routine for the next day. This may happen if the routine is overly ambitious, or closer resources are available but unknown to us. Should a human agent be required to move to a resource, based on the time and their routine, they will move along their path at a speed, that governed by the terrain. For example, movement speed will be faster if the path follows a road, than for traversing land, crops, or bush (the slowest). The speed of movement updates per tick, dependent on the terrain ahead of the individual. The agent will spend differing amounts of time at a resource, depending on the activity, before returning home. For example, an agent collecting water will remain for only a few minutes, as opposed to a schoolchild or farmer who will remain at a resource for several hours. If a human agent responsible for cattle has to make a trip to graze or water cattle, the movement procedure is similar for humans, except that cattle owned by the agent travel with the human agent.

#### Tsetse flies

Tsetse flies start and end each day of the simulation resting in a bushy area within the tsetse zone. Specific movement parameters are described in (Table 3). These include two movement periods in the morning and afternoon, where tsetse move randomly for short bursts, selecting a new direction of travel every time-step, within an 800 m range. The distance decay in Fig 3 provides an impedance for movement of tsetse away from the predefined tsetse zone, such that a proposed movement within or towards the zone is always accepted, and a proposed movement away from the zone is accepted 70% of time. This allows for occasional diffusion of a small number of flies away from the tsetse zone. Although the movement parameter values presented in Table 3 were used throughout the simulation, this is a generalisation, and values are likely to vary in the wild dependent on level of starvation and age of the fly (e.g. teneral vs. mature). Within a single day, various events can occur, including tsetse birth, death, mating, feeding, and infection. These events are discussed below.

**Table 3:** Tsetse movement characteristics.

Parameter	Value	Source/Adapted From
MOVEMENT		
Movement Speed	5 ms^-1^	[51]
Movement Range	800 m radius from daily start location.	[40]
Daily Flight Time	15-30 mins	[52]
Single Flight Period	1-2 mins	[53]
Movement Periods (daily periods where activity can occur)	7 am–11 am, 3 pm–5 pm	[54]
Areal movement impedance	Variable—distance decay, Fig 3	This study

#### Simulation events

The simulation was run for 180 days. 100 repetitions were run across multiple compute nodes, each run taking approximately 65 hours. Repetitions were carried out to account for model stochasticity in the ordering of activities in the human agents, but primarily due to the possibility of a large amount of variation in the timing and spatial distribution of tsetse flies and human infection events. A number of tsetse-oriented events could occur during this period and these processes, interactions, and conditions are described in detail below. A flow chart has been included in the supplementary information (S3 File), describing the model from the point of view of the tsetse per time-step.

#### Tsetse fly target (Identification—Mating and Feeding)

During tsetse morning and evening movement periods, there are two situations that may lead to a tsetse trying to identify a target; namely that an unmated teneral female needs to feed prior to mating, and that an older fly requires to feed. An unmated female will become available for mating if located within the Moore neighbourhood of a male fly. If the female falls within one or more male fly neighbourhoods, that female is considered to have mated.

If a tsetse requires a feed and has not already selected a target, neighbourhood checking occurs as above, except that distances to neighbouring cattle and humans within the Moore neighbourhood are calculated. Possible reactions of tsetse to potential bite victims vary based on how close the fly is to starvation. Findings suggest that humans are repellent to tsetse that are not extremely hungry, and that larger animals that give off more favourable, large odour plumes will be targeted [55]. Human agents are considered possible bite targets only for mature and teneral flies that have unsuccessfully fed on an alternative food source in the current hunger cycle, and would starve the next day should they not acquire a successful feed. Cattle are a suitable and favoured target for any hungry tsetse, and other domestic animals are only considered after two days without a feed, to simulate reduced desirability. Should at least one possible target appear in the neighbourhood of a hungry tsetse the Euclidean distances to each target are calculated, and if one preferable target appears within olfactory range (Table 4), the fly will move directly towards it and attempt to feed. Target preference is exhibited spatially as well as temporally. While humans and cattle are both considered possible targets if a fly is near starvation, a closer human within olfactory range will be ignored in favour of a cow also within olfactory range. If a tsetse moves to a target and feeds unsuccessfully, the fly will select another target in the next tick. This process is repeated (within the tsetse activity time periods) until either the fly feeds or starves. A successfully fed tsetse will then move randomly during the activity periods, potentially undergoing mating, until the 2-3 day interval between feeds is completed, and the search begins again. In addition to simulated tsetse favouring cattle as targets, the probability of a tsetse fly biting a cow that it has identified and moved towards, is 100%. This simplification means that only successful bites are modelled (sustaining the fly for 2-3 days) in place of the potential repeat of partial bites, more likely to be seen in nature. Given that humans are an unfavourable bite target, human bite probability is set to 50% so that within the model, humans are only considered as a bite target as a last resort for tsetse nearing starvation. This difference in bite probability means that humans are less likely to attract tsetse bites than cattle.

**Table 4:** Tsetse feeding parameter values.

Parameter	Value	Source/Adapted From
FEEDING		
Detection Range	140 m	[56–58]
Feeding Interval	2-3 days	[59]
Starvation Period (time between last bite and tsetse death)	Mature—5 days, Teneral—3 days	(Ian Maudlin, pers comm)

Since the density of wildlife is unknown, the probability of a hungry fly feeding on wildlife was estimated and tested by running the model with different parameter values. A daily feed probability of hungry tsetse on wildlife in the tsetse zone of p = 0.35 produced the most stable tsetse population, allowing a population equilibrium to be reached resulting in a 35% chance that a hungry fly will feed on wildlife at the beginning of each day.

#### Infection given a successful tsetse fly bloodmeal

If a tsetse successfully bites an agent, several probability checks are undertaken if either agent is infected, to establish whether the infection has been transmitted, and whether the infection will mature to the salivary glands of the insect and become transmissible (Table 5).

**Table 5:** Infection parameter values.

Parameter	Value	Source/Adapted From
**INFECTION (infected tsetse bites uninfected host)**		
Human Infection Probability	100%	see table footnote *
Cattle Infection Probability	100%	see table footnote *
Incubation period (host)	Cattle—10-14 days; Humans—9-10 days, Large Domestic (non-cattle)—9-10 days, Small Domestic (non-cattle)—6-10 days	[60, 61] Some estimated based on body size.
**INFECTION (uninfected tsetse bites infected host)**		
Midgut infection probability (if susceptible)	First Bite—100%, Future Bites—33%	[62]
Mature infection probability	Male—21% (if midgut positive), Female—4% (if midgut positive)	[4]
Mature infection age reduction (% life expectancy remaining)	Male—to 81%, Female—to 90%	[63]
Incubation Period (tsetse)	17-19 days	[48]

*Using an infection rate of 100% presents a generalisation of a variable that is known to be unpredictable and variable [64]. Studies have shown that transmission success between tsetse and mammalian host can be high (e.g. [65–67]) under laboratory conditions, while Rogers [68] employs a compartmentalised modelling approach which sets the value to 62%. As the presented model, for simplicity, models only successful bites (i.e. no partial bites), sufficient to sustain a tsetse for 2-3 days, the infection rate has been set at a higher value. Average infection numbers produced for a set of simulations which use values of 50%, 60%, 70%, 80% and 90% for this parameter have been included in the supplementary material (S3 Fig), which show little variation when this parameter is set at 70% or above.

As the wildlife reservoir is not modelled as a set of individual agents, each successful wildlife bite is considered to carry an associated infection risk based on the estimated prevalence of *T. b. rhodesiense*. Assuming a prevalence of 0.5%, there is a 0.5% chance that each wildlife bite by a susceptible, teneral fly will acquire a midgut infection, reducing to 0.167% for a susceptible, non-teneral fly. Rates of maturation in the fly are identical to those described for agent bites.

#### Tsetse fly births

Once a female tsetse has mated, she will start depositing offspring 18 days from the date of mating, and every 10 days thereafter until death [43]. There is an equal chance of each tsetse offspring being male or female. On emergence, tsetse are defined as teneral (unfed) until their first bloodmeal. Males have a longer pupal period than females [43, 69] and so for each larva deposited during the simulation, there is a 35 day pupal period in males and 30 day period in females, represented as a period of inactivity. Pupation is temperature sensitive with pupal periods decreasing with increasing temperature, and with 23–25°C considered optimum [69, 70]. Mortality in pupae in the wild show a 26% chance that each pupa will die before developing into an adult [71]. Should a pupa become an adult, a teneral period is included prior to the first feed, represented by a shorter time before starvation without feeding. For the first 35 days, 75 teneral flies are added to the model at random locations within the initial tsetse zone to aid birth rate continuity. This is representative of any pupae present at the start of the simulation that develop into adults.

#### Tsetse fly deaths

Death can result from pupal mortality, starvation, or the exceeding of a scaled version of the mortality rate produced by Hargrove et al. [15] for male and female tsetse. These predicted mortality rates for tsetse were scaled down to dissociate the impact of starvation as this is modelled independently. Through scaling, the aim was to estimate a probability of dying by age, if the fly agent does not die of starvation. As with the probability of a fly agent gaining a feed from wildlife, the value of this scaling parameter had to be estimated, and the product of parameter testing suggested that the most stable tsetse population would be produced using a scaling factor of 55%.

Starvation occurs if a tsetse tries and fails to feed before a given period of time has elapsed. The starvation element is stricter for teneral flies (3 days instead of 5 days) highlighting their increased vulnerability and reduced flight strength.

Given that estimations were used for both the probability of a hungry tsetse gaining a feed from wildlife in any given day, and the degree of scaling required for the mortality rate, sensitivity analysis was carried out for a range of values of each of these parameters (see S4 Fig). The values identified previously produced the most stable tsetse population across the simulation period, and are therefore used in the production of the main results.

## Results

### Tsetse Fly Population

**Fig 4:**
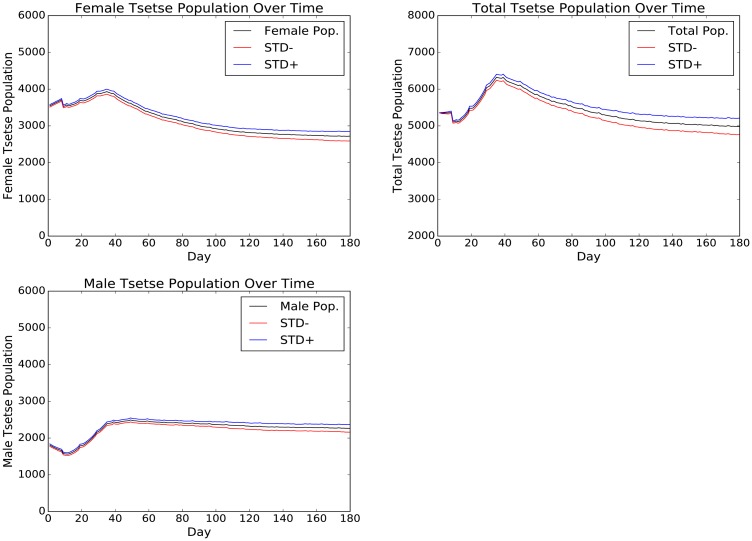
Average tsetse population over a six month period. STD- and STD+ refer to the ranges of the standard deviation.

Results for the tsetse population across a six-month period are shown as an average of the 100 simulation repetitions, using standard deviation as a measure of stability (Fig 4).

The total tsetse population shows an initial period of fluctuation, including an initial sharp drop from the initial population of 5,250 flies. The population reaches a peak of 6,200 at around day 40, before steadily decreasing to near-stability at around day 110 (population of around 5,200 flies). The male population appears more stable throughout, with much of the variability in the overall population being driven by the female tsetse. Although starting with a 2:1 female-to-male ratio as suggested in the literature [41, 42], by the time equilibrium is reached, this ratio is approximately 4:3.

### Tsetse Mortality

The prominence of different modes of death for tsetse varies as the simulation progresses (S5 Fig). Before day 40, a large number of deaths are attributed to adult starvation and natural causes. Thereafter, adult starvation reduces, with natural death being the primary cause of around 50 tsetse deaths per day. Pupal mortality is the next highest cause of death, followed by starvation of teneral flies once the simulation has become stable.

The average ages at which tsetse die throughout the simulation, regardless of cause is shown in S6 Fig. Despite modification of the published mortality rates to allow the modelling of starvation and puparial deaths separately, the death rates of male and female tsetse follow a similar trend, with a significant decrease in the number of male tsetse living beyond 40 days, as the driving mortality rate reaches 20% per day.

### Tsetse Fly Feeding

**Fig 5:**
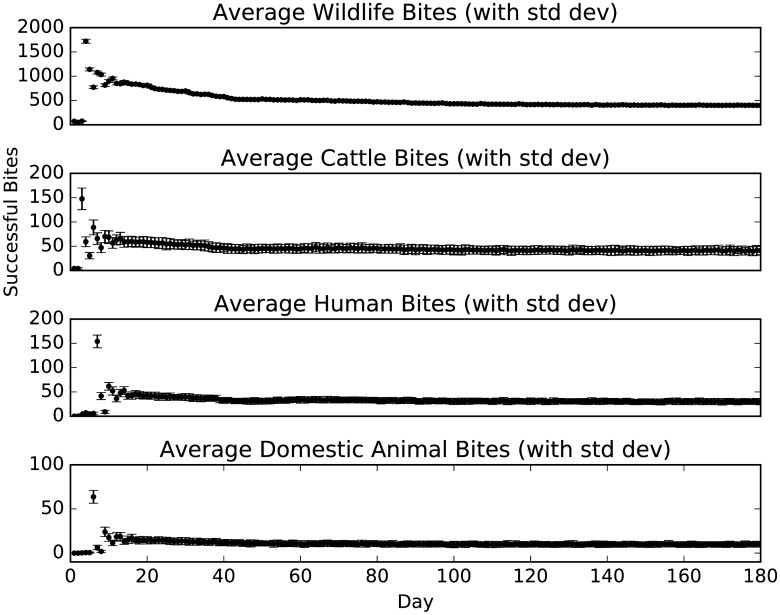
Successful tsetse blood meals, separated by host type.

The distribution of successful host bites by day is dominated by wildlife feeds throughout, with 400 to 500 bloodmeals per day once the simulation has reached equilibrium at approximately day 60 (Fig 5). Cattle, other domestic animals and human hosts provide less than 50 feeds per day on average. Although it is expected that cattle are a more favourable bite target than humans, the greater human population, and a large number of households without cattle, appear to negate the expected difference to some degree, with an average of 46 bites on cattle per day, compared with an average of 33 human bites. The average number of daily bites on other domestic animals is 11.

### Locations of Acquisition of Infection

**Fig 6:**
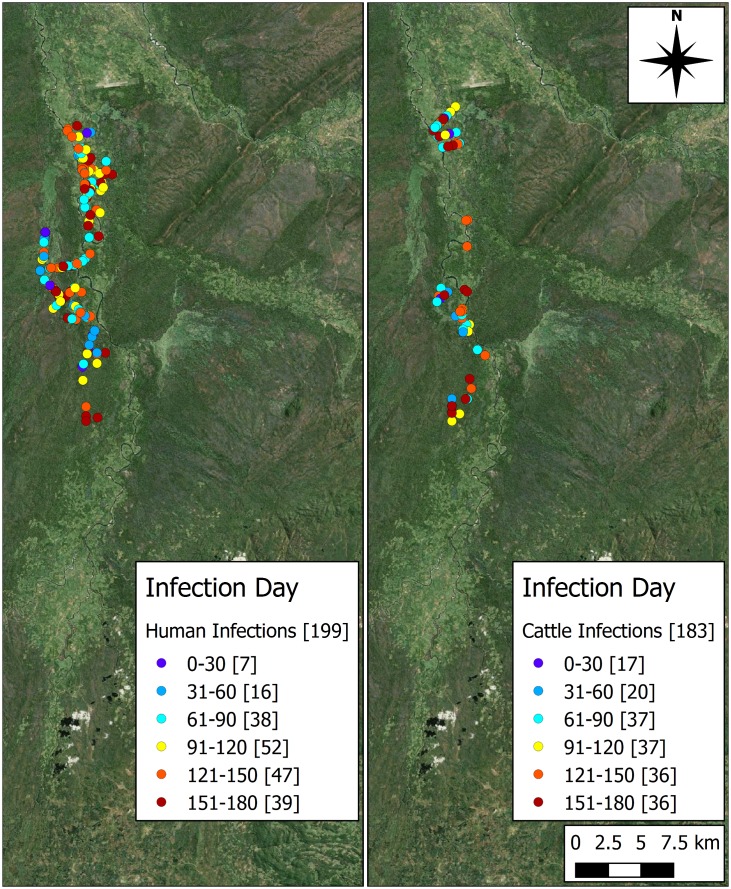
Spatial representation of human (left) and cattle (right) infections by day (aggregate of 100 repetitions). [Number per bin]. Produced using Landsat 7 imagery available from USGS.

The aggregated spatial distribution of rHAT infections by day for both cattle and human hosts, is shown in Fig 6. Across the 100 repetitions, the mean number of human infections is 1.99 (S.E. 0.245), while the mean number of cattle infections is 1.83 (S.E. 0.183). Cattle infections are localised, while human infections are more dispersed, mostly in the areas with fewer cattle infections. Both sets of data show an increase in new infections as the simulations progress.

While the number of infections is similar between human and cattle agents, the incidence rate across the area varies given the difference in population size between the two hosts. The approximate incidence of *T. b. rhodesiense* infection in cattle is 0.588 per 1000 cattle-years (S.E. 0.059) and the approximate incidence of human rHAT is 0.124 (S.E. 0.015) per 1000 person-years.

### Human Infections by Demographic, Activity and Tribe

Within the simulation, infected agents can be interrogated to acquire information about their characteristics (e.g. age), and the circumstances of their infection. Table 6 shows a slightly higher rHAT incidence amongst females than in males amongst the human agents that became infected across the 100 repeat simulations. The most susceptible age group was 5–10 years of age, which could be due to the schooling activities, unique to the younger age group. Older children (10–18 years) were more likely to have a diverse range of activities in addition to schooling, including farming. The incidence rate for humans from non-cattle keeping households was considerably higher than for households with cattle ownership.

**Table 6:** Average incidence of human infections by age, gender and presence of household cattle.

Human Infections	(Approx. Incidence Rates reported as per 1000 person-years)							
Age	IR	SE	Sex	IR	SE	Cattle in the Household?	IR	SE
0–5	0.013	0.006	Male	0.111	0.017	Yes	0.079	0.017
5 to 10	0.239	0.041	Female	0.137	0.016	No	0.134	0.017
10 to 18	0.185	0.030						
18 to 60	0.103	0.016						
60+	0.092	0.034						

The predominant activities undertaken when acquiring an infection are travelling to and from school, tending to crops, and while acquiring water from the river or a borehole (Table 7). No infections are acquired when leaving the village with cattle, while only 4.3% occur when human agents are resting within the village area.

**Table 7:** Percentage of human infections by activity.

Activity	% of human Infections	SE
School	**24.6**	4.145
Resting	4.3	2.137
Market	0	0
Firewood	8.3	2.905
Farming	**29.6**	4.233
Water	**33.3**	4.359
Graze Cattle	0	0
Water Cattle	0	0

While incidence is generally low across tribes (Table 8), the results suggest that immigrant tribes may be at the greatest risk of infection. These tribes make up less than 30% of the total population.

**Table 8:** Infected human ethnicity (Approx. Incidence Rates reported as per 1000 person-years).

Infected human ethnicity (Approx. Incidence Rates reported as per 1000 person-years)			
Ethnicity	Description	IR	SE
**Chewa**	Immigrant; from Chipata/Katete	0.332	0.045
**Kunda**	Indigenous; poor; keeping mostly goats and sheep traditionally	0.083	0.014
**Ngoni**	Immigrant; from Chipata/Katete	0.174	0.086
**Nsenga**	Immigrant	0.011	0.011
**Bemba**	Immigrant; northern Zambia and Copperbelt	0.353	0.155
**Lenje**	Immigrant	0.727	0.512

### Infection by Home Location and Cattle Ownership

**Fig 7:**
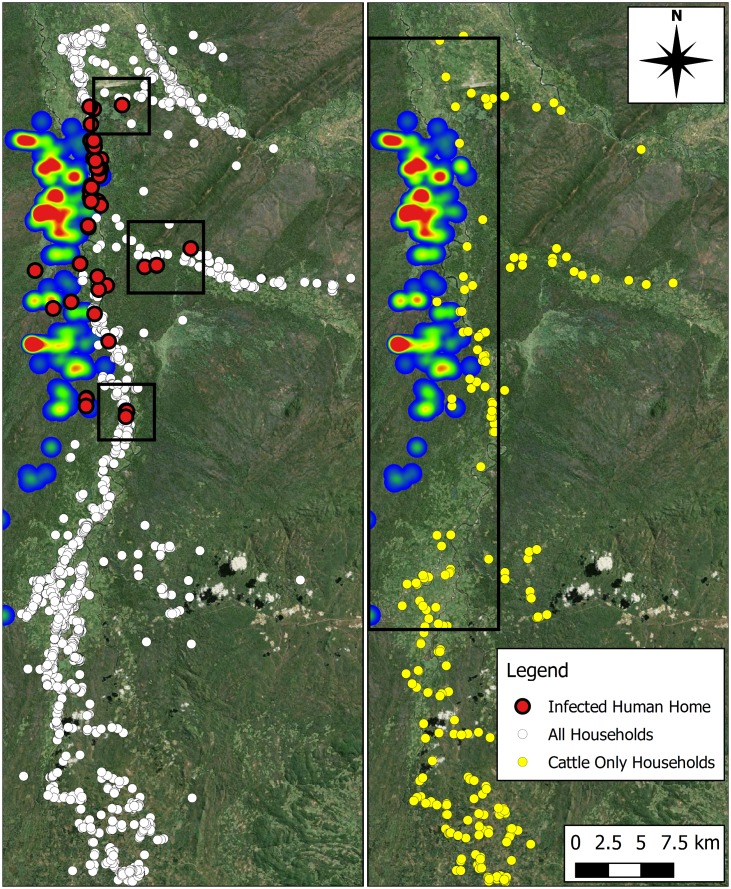
(Left) Illustrates the home locations of human agents which acquire a rHAT infection (199 rHAT infections in total). The three boxes show village locations away from the tsetse zone, indicating that some agents become infected as a result of converging on the tsetse zone for resources, which exposes them to the biting hazard. White circles illustrate the locations of all households within the simulation. (Right) Distribution of cattle owning households (circles) overlain with the estimated tsetse distribution. Box containing the tsetse fly zone and surrounding area is home to 547 (yellow circles) of the 2,925 cattle in the region. Produced using Landsat 7 imagery available from USGS.

An illustration of the locations of households inhabited by people who become infected during their daily routine, overlain on the tsetse KDE map is shown in Fig 7. The spatial distribution of villages affected by infection is wider than the distribution of the locations of infection occurrence as seen in Fig 6. This highlights the distance travelled by some agents for resources, with villages being approximately 5–10 km from the edge of the tsetse zone (see Fig 7).

The infection rate among cattle owners compared to people without cattle is noticeably lower. No human infections were acquired by agents grazing or watering cattle agents. One possible factor influencing this interaction is the distribution of cattle. Approximately 20% (547 of the 2,925) of the cattle population belong to households in close proximity to the tsetse fly zone (Fig 7).

### Activity, Resource Availability and Exposure

**Fig 8:**
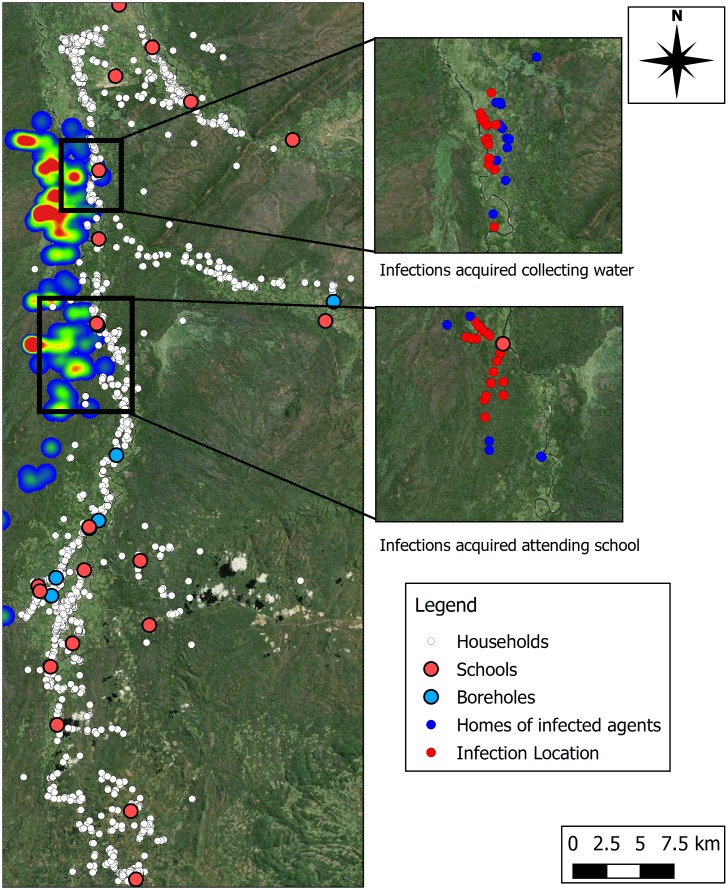
(Left) Locations of households, boreholes and schools across the study area, overlain on the tsetse fly zone. (Right) Two sites of interest highlighting homes of infected agents, and locations of infection for those agents when collecting water (above), and attending school (below). Produced using Landsat 7 imagery available from USGS.

Fig 8 presents information illustrating the locations of infections (and home locations of those infected) for two of the activities that produce the most infection in the model: school attendance and water collection, at two sites of interest. For infections acquired when collecting water the image illustrates that, with the absence of a local borehole, households here are particularly exposed to tsetse flies in the relatively short walk between home and river location. Conversely for school attendance, trip frequency is lower, and infection through this activity can only be transmitted to a fraction of the demographic. However, the relative sparsity of schools in regions within (and adjacent to) the tsetse fly zone results in increased trip distances in this area (up to 4.5 km in the area shown). Here, some of the households with inhabitants that become infected during trips to schools are situated at the edge, or outside of the previously mentioned tsetse zone.

### Tsetse Fly Infections

**Fig 9:**
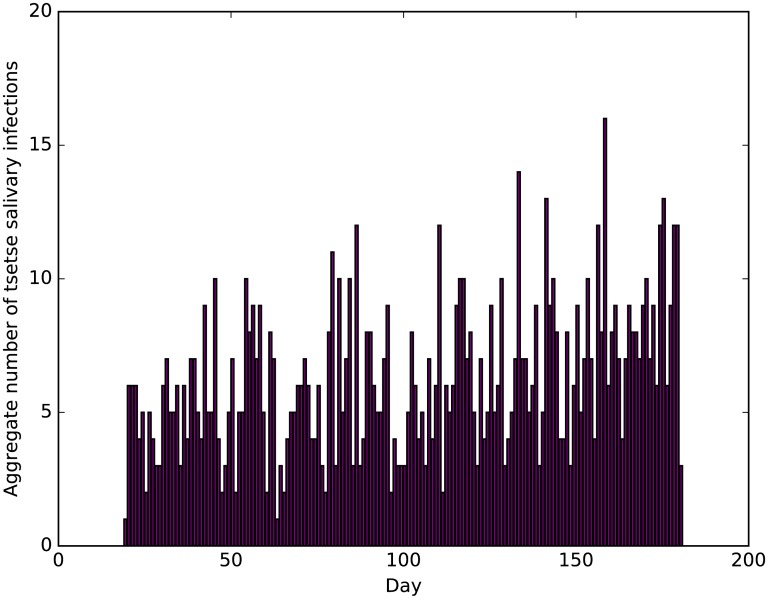
Aggregate number of tsetse salivary infections by day.

Progression of midgut and salivary gland infections in the tsetse population suggests that, on average, one new midgut infection is acquired within the tsetse population per day (S7 Fig). The aggregate number of salivary gland infections in the tsetse population through time, across all repetitions, suggests more infections are maturing in the latter stages of the simulation (Fig 9).

## Discussion

Through the construction of a multi-host ABM, incorporating detailed characteristics and complex mechanisms such as the tsetse life-cycle and real-world human population dynamics, this research presents a plausible environment for the investigation of rHAT transmission in the study area. Simulation results suggest that school attendance and water collection are high risk activities, and that immigrant tribes are at a greater risk of rHAT infection than non-immigrant tribes. These results suggest that reducing the average distance between villages and schools, while increasing the provision of borehole water resources to villages in close proximity to the tsetse fly zone, would aid a reduction in exposure to the vector, and therefore reduce transmission. The relationship observed between tribe and human infections may be small, but the higher rates of infection in the Bemba and Lenje tribes is curious. These migratory tribes have the smallest population in the study area but are more likely to settle in increasingly marginal land, potentially closer to the wildlife interface, which may increase exposure to infected tsetse and therefore rHAT. The results also suggest that future uncontrolled migration into the area may result in an increase in rHAT transmission.

The ABM was applied to model a tsetse population, reaching a degree of stability in all modelled characteristics within 6 months. Modelled characteristics include tsetse population size, feeding behaviour, mode of death, establishment of infection and distributions of ages of death for both tsetse sexes, that correspond to predicted mortality rates [15]. The resultant model behaviour was largely consistent with the literature, with the exception of an increased ratio of males to females between the start and stable tsetse population. While the higher proportion of females was suggested in the literature (2:1, female-to-male) [41, 42], and was used as the start value for the model, stability in the tsetse population was reached at the 4:3 ratio. Fig 4 illustrates how the model was fitted. The effect of this gender shift could produce a greater rate of transmission than models using a static 2:1 ratio, given that male tsetse are more susceptible to salivary gland infection [4]. This is unlikely to have had an effect on the results presented in this research, considering the short simulated time and low infection rates, however, it is an important outcome to consider over longer periods and in the real world, with future empirical study required to further investigate this tsetse gender ratio in the wild.

The simulation results suggest that attending school, collecting water and farming are the most risky activities to human agents, accounting for over 90% of acquired infections. Causal factors could include: location of the resources and the frequency with which they are accessed (e.g. river adjacent to the tsetse habitat), the amount of time spent at the resource (e.g. farming) and the relatively long walks to a resource due to sparse distribution (e.g. of schools). Fig 8 highlights how trips in the area with very different characteristics can produce a similarly high risk of infection. Despite the area highlighting a high number of water collection infections being at the edge of the tsetse fly zone, and the trip time being short, the high frequency of trips to collect water may be driving the number of infections. Conversely, the sparsity of schools in and around the tsetse fly zone not only increases exposure to the tsetse fly by requiring long walks in the region, but also widens the influence of the tsetse zone, as individuals enter the region to attend school. As such, an individual’s own behaviour increases their exposure to the tsetse fly and, thus, increases their risk of infection. For school trips in particular, the time period of trips in the morning and afternoon coincide with the activity periods outlined for tsetse flies, providing an additional increase in risk. Another possible cause is the absence of cattle in these resource trips—a more preferable bite target than humans. Another explanation is that the location of the home in which the agents reside drives disease risk.

The best protection from rHAT risk is distance from the vector, with the majority of cattle owning households situated in the south of the study area. The average number of daily bites on cattle is greater than that for humans, offering a potential dilution effect related to the cattle due to their higher desirability as a bite target. Although infrequent, some people acquiring infections may live approximately 5 or 6 km away from the edge of the tsetse fly zone, highlighting the large distances that some people live away from sparser resources such as markets and schools, which can heighten exposure. Information such as this could be used to aid location targeted mitigation strategies aimed at resource provision and the adaptation of people’s routines (i.e. to make high exposure journeys in tsetse resting periods) in the future.

The ABM suggests certain demographic and behavioural characteristics can vary the risk of acquisition of rHAT in the study region. The locations of homesteads and the locations of visited resources are of high importance, but several less obvious relationships, such as cattle ownership and immigrant tribe status, may drive heightening disease risk. Further research using this ABM framework will test a series of what-if scenarios, including hypothetical situations which change and increase the cattle population, add resources, and assess the implications of an increasing number of marginal, immigrant settlements on both host infection and the tsetse population.

The ABM presented in this paper offers a means of developing mitigation strategies for the area and for One Heath education. The fine spatial scales involved in the modelling process, allow incorporation of information on demography, and the size of the area is representative of the scale over which potential mitigation strategies may be implemented.

The addition of seasonality within the ABM would permit the simulation to be run over longer time periods. A further development for the ABM will be to incorporate temperature and rainfall data and develop the tsetse agent class to be sensitive to changes in both. More detailed wildlife data added to the simulation could aid accuracy in future development, should this become available.

The model has been developed in as generalised a way as possible in terms of computer coding and, therefore, is not restricted to the study of rHAT in Zambia. While tailoring would be required to transfer the model to another location (census data, human movement information, and localised tsetse survey data), this effort would not be applied to the model itself, with any location specific data imported from external files rather than being embedded in the model. In the absence of these data, the model would still run with estimates (estimated human and tsetse populations, village locations and known resource locations from satellite or aerial sensor imagery, and human movement paths based on A* pathfinding) with the caveat that there would be greater uncertainty within the model. Similarly, although more expensive in terms of build time, the use of a class system for the tsetse agents means that, given the correct information profile of the vector, the model could be used to explore different vector-borne diseases. The future application of the model to a similar site of interest in Zimbabwe will allow further exploration of model parameter values, and help identify the level of generalisation presented within the current choices.

Model validation with respect to infection rates in the region is not possible at this time due to the sparsity of data. Furthermore, the estimated high levels of under-reporting make estimates of incidence a difficult task. However, through careful construction with guidance from experts and the literature, the incorporation of complex mechanisms, and the conducting of thorough sensitivity analysis for unknown parameter values, the ABM can be seen as the development of a plausible “universe” where the transmission system can be explored. With the development of our understanding being one of the fundamental purposes of model construction, the ability to explore the system at such a fine scale through the ABM can be seen as a primary use. Whether this is the modification of the environment or agent populations, perturbations to the model can be applied to see how modification affect outcomes, potentially helping to identify unknowns, and provide a stimulus for further investigation. As such, a lot of freedom exists to explore the modelled system, and perturbations applied to it. Future research will consider modification of the environment (e.g. the removal of bush as tsetse resting sites), and the population in the model (e.g. an increase number of migrants), to help generate hypotheses about the system’s response to these perturbations.

Further investigation will consider the degree of detail required to produce similar results to those presented here. An important methodological question remains surrounding the required level of parameterisation, and the effect of generalising model inputs, structure and parameters on the model output provides an important question to investigate in future. Importantly, the existence of a detailed ABM such as here provides a starting point for generalisation experiments, and allows such an investigation to be undertaken.

## Conclusion

The work presented here has shown that it is possible to produce a plausible, detailed ABM for rHAT transmission at a fine spatial scale. The ABM fitted here is the first to model the tsetse vector at the individual level. Such a modelling technique can be used in conjunction with more traditional techniques such as compartmentalised approaches, to test hypotheses and ask questions of the transmission system. Through the identification of possible spatial, demographic and behavioural characteristics which may have differing implications for rHAT risk in the region, the ABM has produced output that could not be readily produced through compartmentalised approaches and, as such, has generated hypotheses that can be tested (through the ABM), including possible mitigation strategies at the regional level.

## Supporting Information

S1 Fig Typical village homestead (Image: Simon Alderton). (TIFF)

S2 Fig Land classification and pathfinding images. (A) A section of the 11 m resolution land classification image, illustrating bare land areas around the airport (pink), cropland (yellow), bush/forest (green), and finer scale digitised features including roads (gold) and river (blue). (B) Example path produced using the A* algorithm and land classification between arbitrary points. Arbitrary points were used to emphasise how the algorithm diverts the path around a prominent obstacle; in this case, the river itself (Produced using Bing aerial imagery), after [39].
(TIFF)

S1 File Summary data from the human routine questionnaire. (DOCX)

S2 File Results illustrating the average number of infections over a 6-month simulation for 25 combinations of human and cattle infections (25 repeats). (PDF)

S3 File Model routine for a single tsetse per time-step. (PDF)

S3 Fig Sensitivity analysis showing the impact of changing the tsetse-to-host infection probability, given a successful bite. (TIFF)

S4 Fig Sensitivity analysis highlighting the response in the tsetse population to different values of wildlife feed probability (daily per unfed tsetse), and non-starvation mortality rate scaling value. 9 combinations were run for 25 repetitions each. The highlighted value set is the one chosen for the main analysis.
(TIFF)

S5 Fig Average number of tsetse deaths over time, separated by cause of death. (TIFF)

S6 Fig Tsetse death ages by gender. Average male (top) and female (bottom) tsetse deaths against age. The inset images illustrate the mortality rates from the literature [15] used to shape the non-starvation death rate.
(TIFF)

S7 Fig Average midgut and salivary gland infections with standard deviation (100 simulations). (TIFF)
