# Supplementary figures and images for: A Multi-Host Agent-Based Model for a Zoonotic, Vector-Borne Disease. A Case Study on Trypanosomiasis in Eastern Province, Zambia
Source: PLoS Negl Trop Dis. 2016 Dec 27;10(12):e0005252. doi: 10.1371/journal.pntd.0005252 (PMC5222522; doi:10.1371/journal.pntd.0005252)

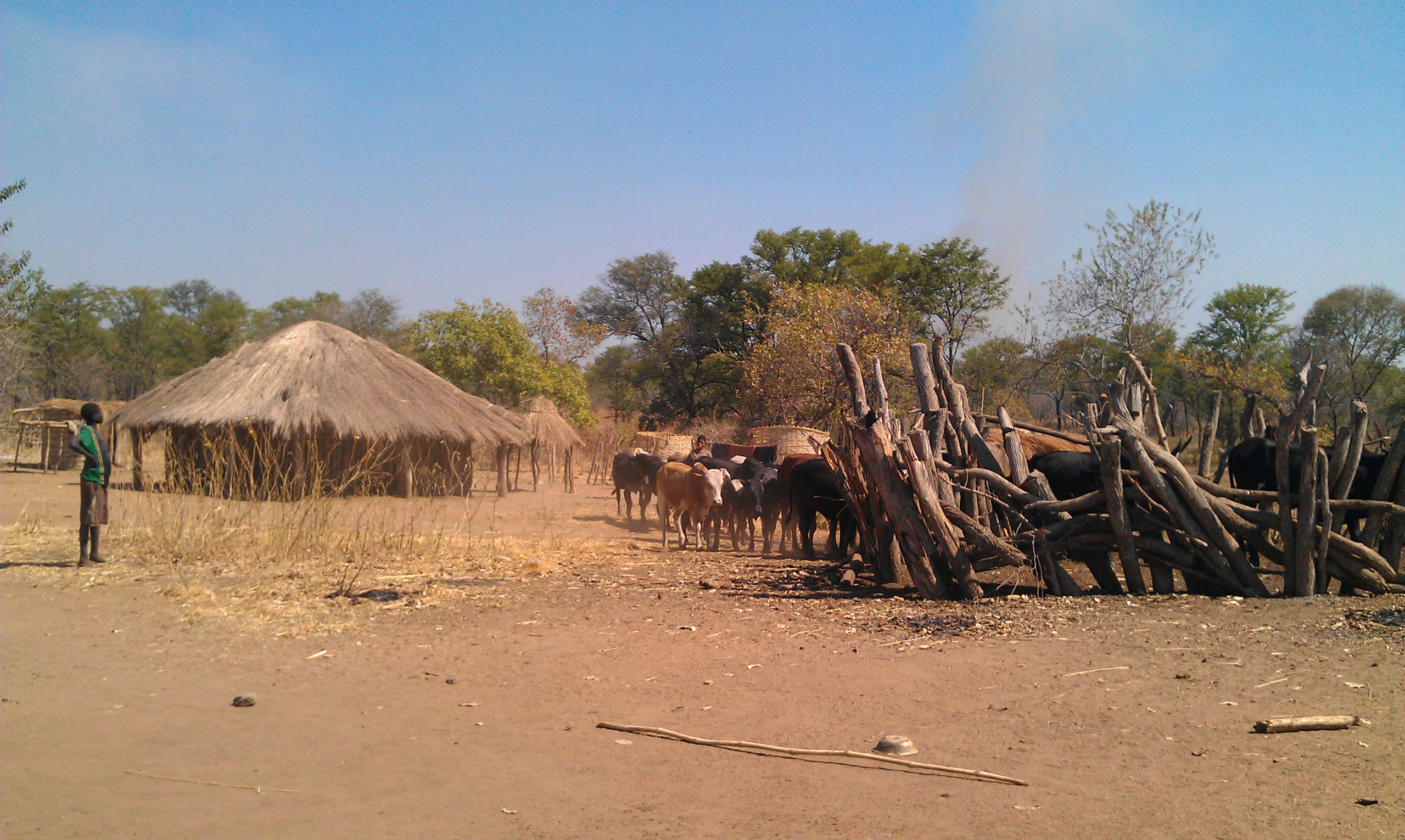

Supplement: S1 Fig — (TIFF) [file pntd.0005252.s001.tiff]

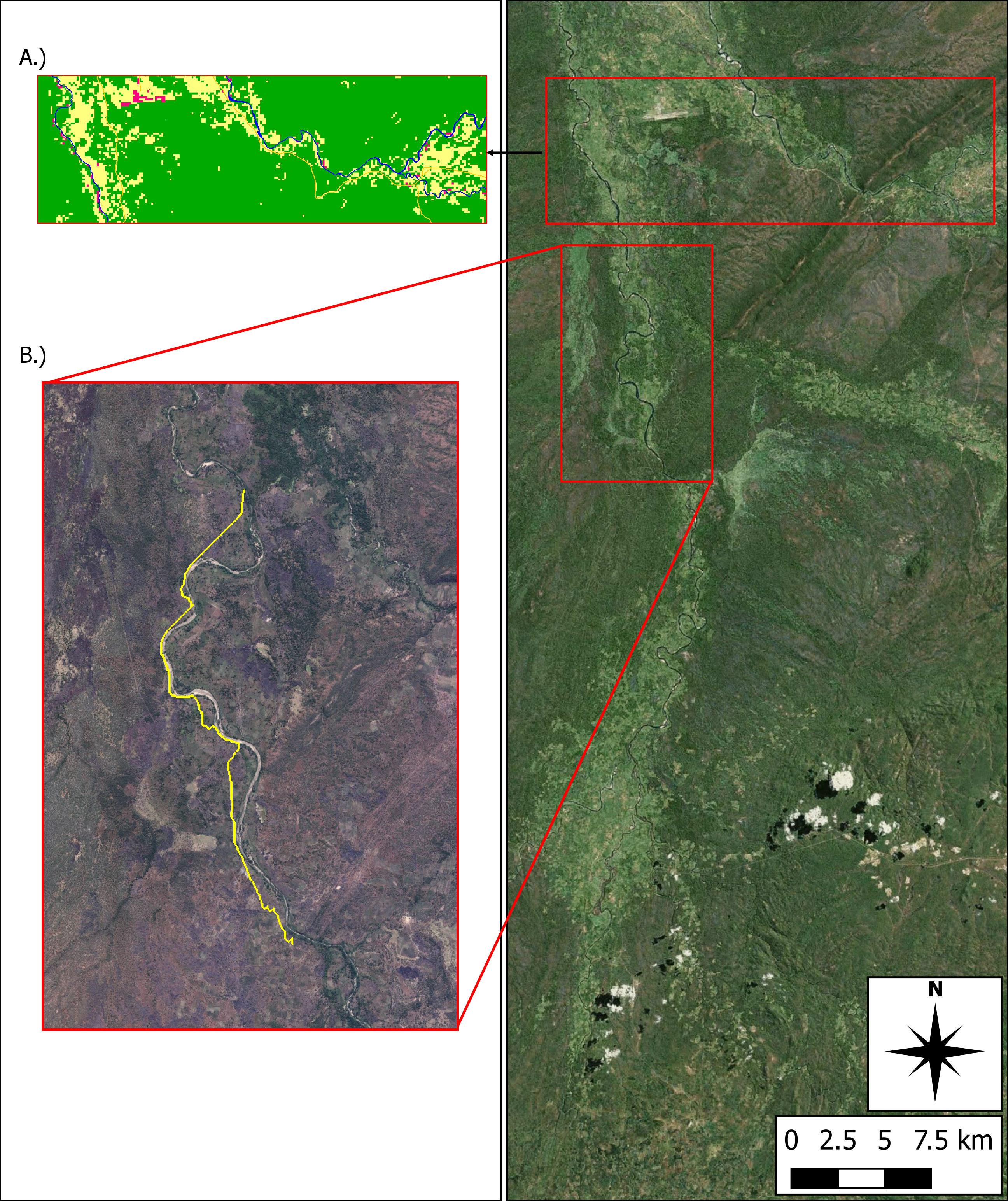

Supplement: S2 Fig — (A) A section of the 11 m resolution land classification image, illustrating bare land areas around the airport (pink), cropland (yellow), bush/forest (green), and finer scale digitised features including roads (gold) and river (blue). (B) Example path produced using the A* algorithm and land classification between arbitrary points. Arbitrary points were used to emphasise how the algorithm diverts the path around a prominent obstacle; in this case, the river itself (Produced using Bing aerial imagery), after [39]. (TIFF) [file pntd.0005252.s002.tiff]

Cattle Infections

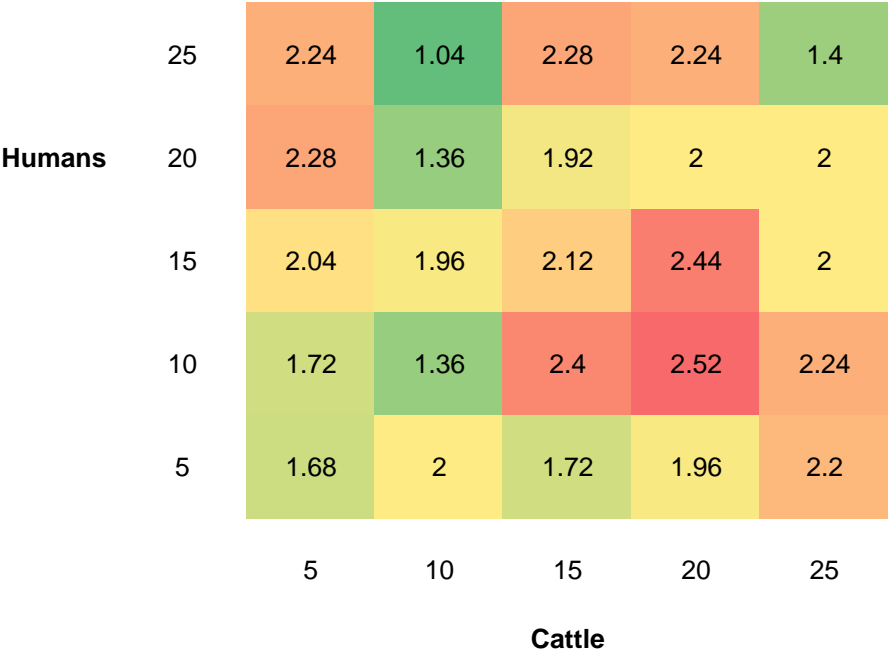

Human Infections

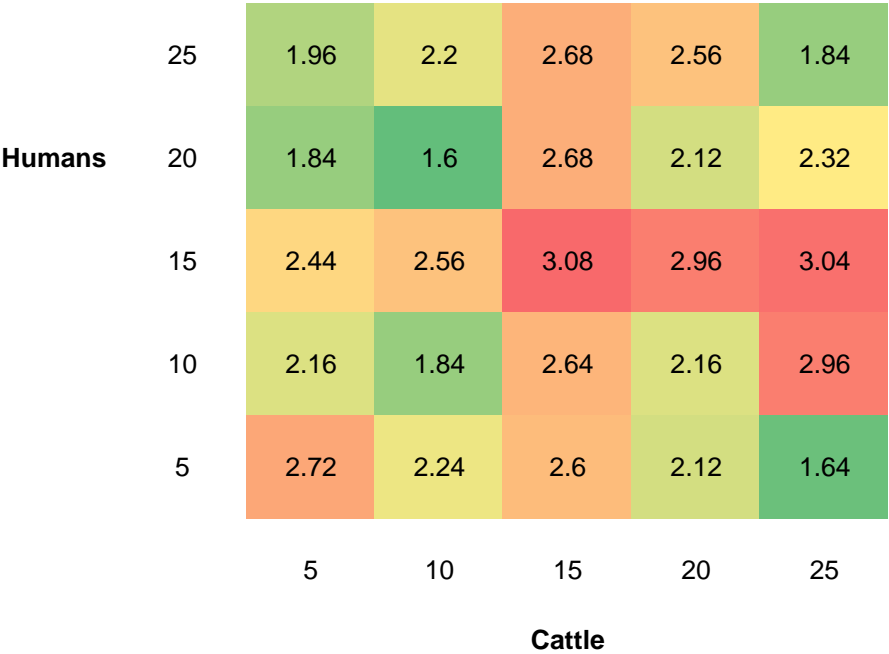

Supplement: S2 File — (PDF) [file pntd.0005252.s004.pdf]

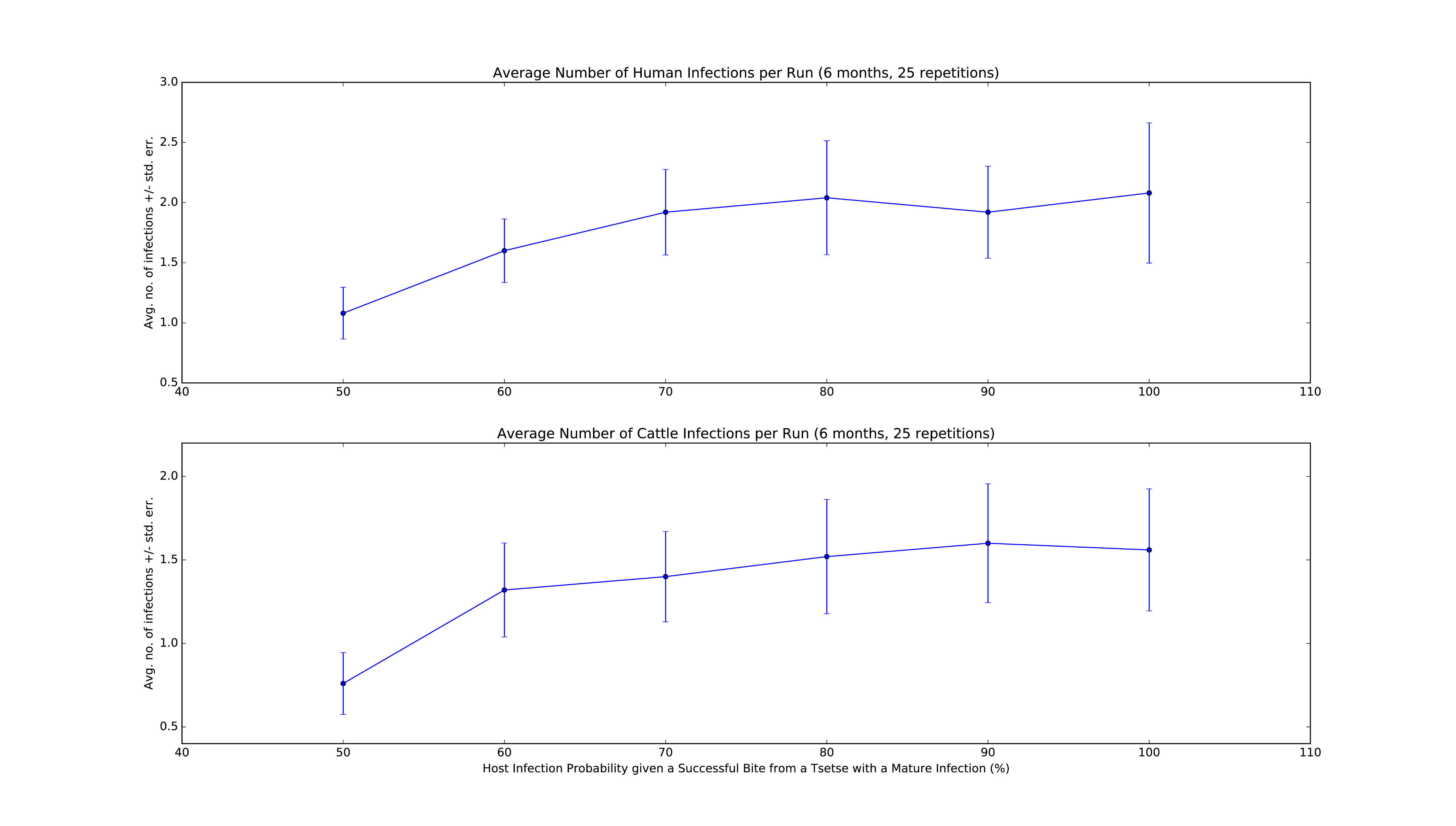

Supplement: S3 Fig — (TIFF) [file pntd.0005252.s006.tiff]

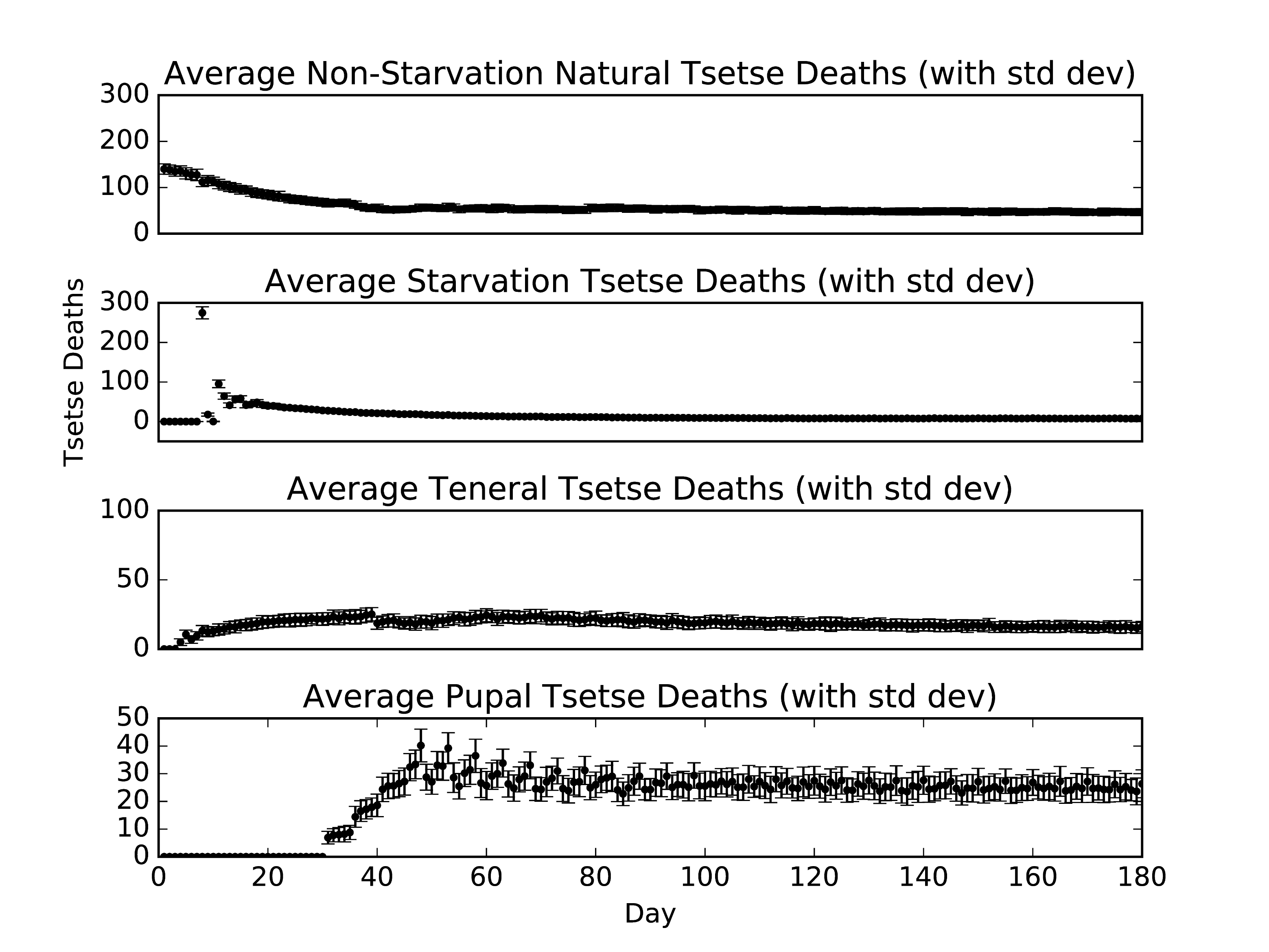

Supplement: S5 Fig — (TIFF) [file pntd.0005252.s008.tiff]

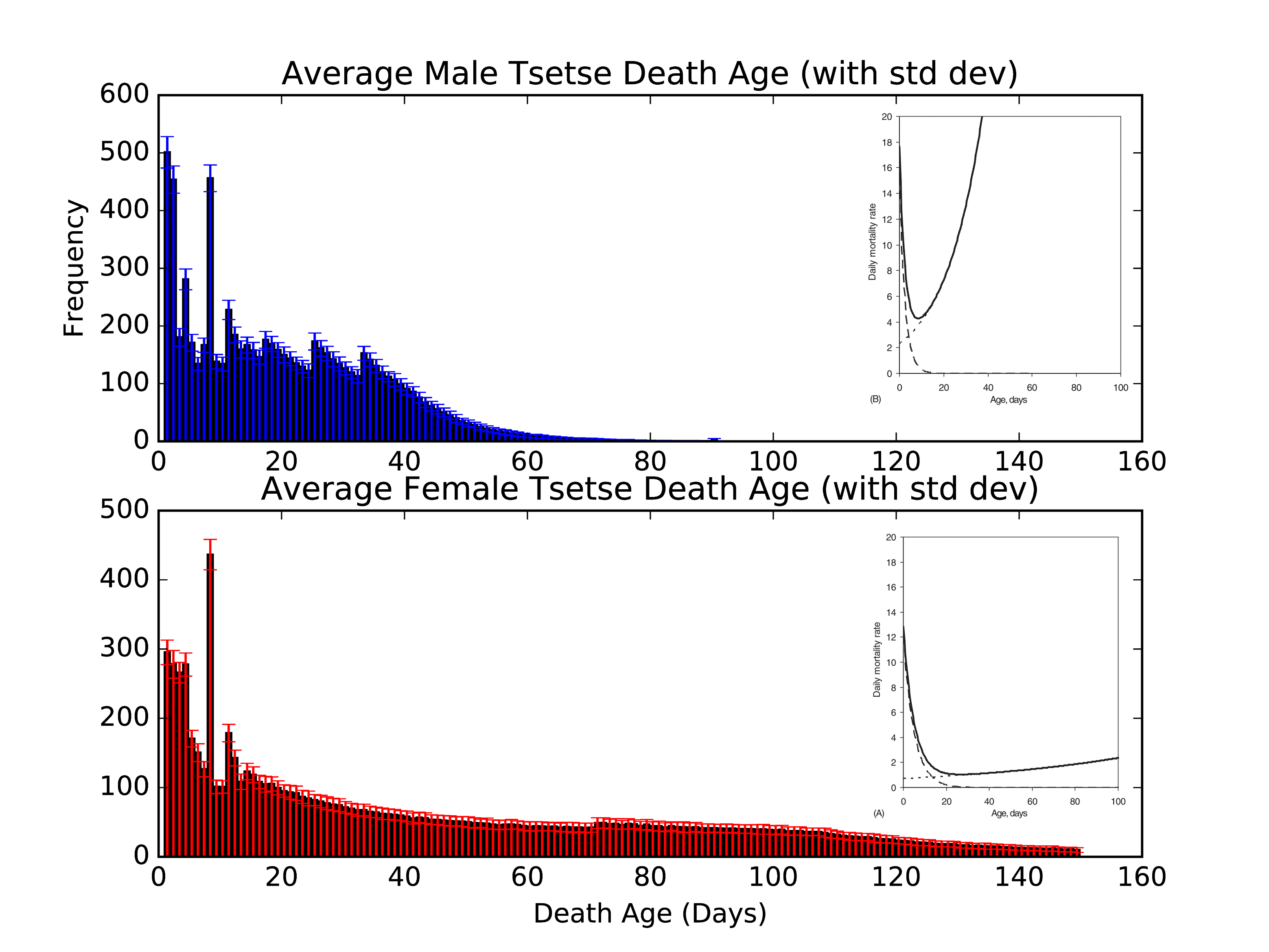

Supplement: S6 Fig — Average male (top) and female (bottom) tsetse deaths against age. The inset images illustrate the mortality rates from the literature [15] used to shape the non-starvation death rate. (TIFF) [file pntd.0005252.s009.tiff]

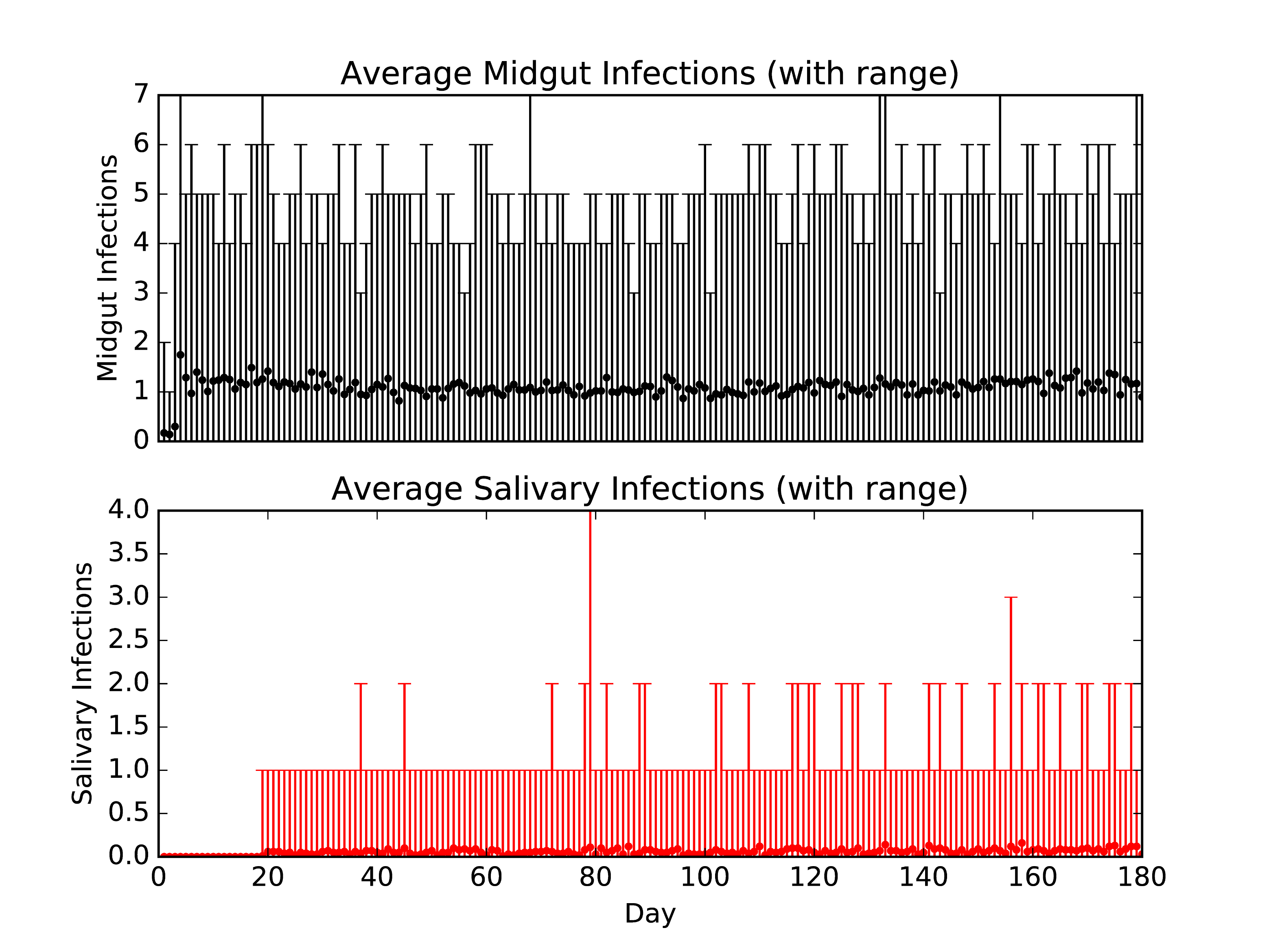

Supplement: S7 Fig — (TIFF) [file pntd.0005252.s010.tiff]
