# Supplementary material for: A Multi-Host Agent-Based Model for a Zoonotic, Vector-Borne Disease. A Case Study on Trypanosomiasis in Eastern Province, Zambia
Source: PLoS Negl Trop Dis. 2016 Dec 27;10(12):e0005252. doi: 10.1371/journal.pntd.0005252 (PMC5222522; doi:10.1371/journal.pntd.0005252)
Supplement: S1 File — (DOCX) [file pntd.0005252.s003.docx]

**Data Collected on Individual’s Routines**

**Respondents: 126**

**Gender:**

Male: 45

Female: 80

No response: 1

**Age:**

Median: 32, Mean: 36, Min: 9, Max: 76.

No response: 2.

Figure 1 - The data represents preferential sample, asking potential respondents who were available within each village at the time of data collection. As a result, the number of young respondents is low, and it is expected that these children were attending school. However, information concerning their school attendance was captured from other respondents by proxy, with questions included which queried the attendance of school by other members of the household.

**Role:**

Farmer: 107

Pupil: 4 (**although other school attendees are listed as farmers**)

No response: 3

(Others include a brick layer, hospital cleaner, carpenter, and some native names for occupations).

In total, 124 routines could be created to resample from as a result of 2 sets of responses having missing data for 1 or more resources. Routines were divided into three age categories for both males and female: 6-17, 18-59 and 60+. No data was collected on behalf of children under the age of 5.

Table 1 - The percentage of each age/gender group’s routines that contained each resource. N.B. For children of school age, both farming and school attendance appear frequently. This can be attributed to children often attending school for half days and then assisting with work in the fields.

| **Routine** | | **Daily/regularly** | | | | | | **Monthly** |
| --- | --- | --- | --- | --- | --- | --- | --- | --- |
|  |  | **Water %** | **Charcoal %** | **Farm %** | **water cattle %** | **graze cattle %** | **Schools %** | **Market %** |
| Male | 0-5 |  |  |  |  |  |  |  |
| Male | 6-17 | 80 | 60 | 80 | 40 | 60 | 100 | 80 |
| Male | 18-59 | 81 | 100 | 86 | 19 | 11 | 0 | 56 |
| Male | 60+ | 100 | 100 | 25 | 0 | 0 | 0 | 50 |
|  |  |  |  |  |  |  |  |  |
|  |  |  |  |  |  |  |  |  |
| Female | 0-5 |  |  |  |  |  |  |  |
| Female | 6-17 | 100 | 100 | 94 | 50 | 44 | 94 | 50 |
| Female | 18-59 | 98 | 100 | 92 | 15 | 15 | 0 | 69 |
| Female | 60+ | 100 | 82 | 55 | 9 | 0 | 0 | 27 |

Table 2 - For trips that are carried out more than once a day (e.g. water and charcoal collection) the frequencies of trips made, along with the associated time periods, was recorded.

|  | **Water** |  |  |  | **Charcoal** |  |  |  |
| --- | --- | --- | --- | --- | --- | --- | --- | --- |
| **How frequent?** | **%** | **What times?** | **%** |  | **How frequent?** | **%** | **What times?** | **%** |
| Several Per Day | **79** | Morning | **48** |  | Several Per Day | **13** | Morning | **72** |
| Once Per Day | **13** | Lunchtime | **10** |  | Once Per Day | **62** | Lunchtime | **2** |
| Rarely | **2** | Afternoon | **32** |  | Rarely | **24** | Afternoon | **23** |
| Not Recorded | **7** | Evening | **10** |  | Not Recorded | **2** | Evening | **3** |
