# Supplementary material for: A Multi-Host Agent-Based Model for a Zoonotic, Vector-Borne Disease. A Case Study on Trypanosomiasis in Eastern Province, Zambia
Source: PLoS Negl Trop Dis. 2016 Dec 27;10(12):e0005252. doi: 10.1371/journal.pntd.0005252 (PMC5222522; doi:10.1371/journal.pntd.0005252)

Tsetse time-step flow-chart

Daily Probability Checks:

(Take place at start of day)

Death

Adult/teneral starvation

Non-starvation death (scaled mortality rate)

Pupal death

Feeding

Wildlife feed probability if unfed

Reproduction

First offspring after 10 days (if mated female)

Further offspring every 18 days (if mated female)

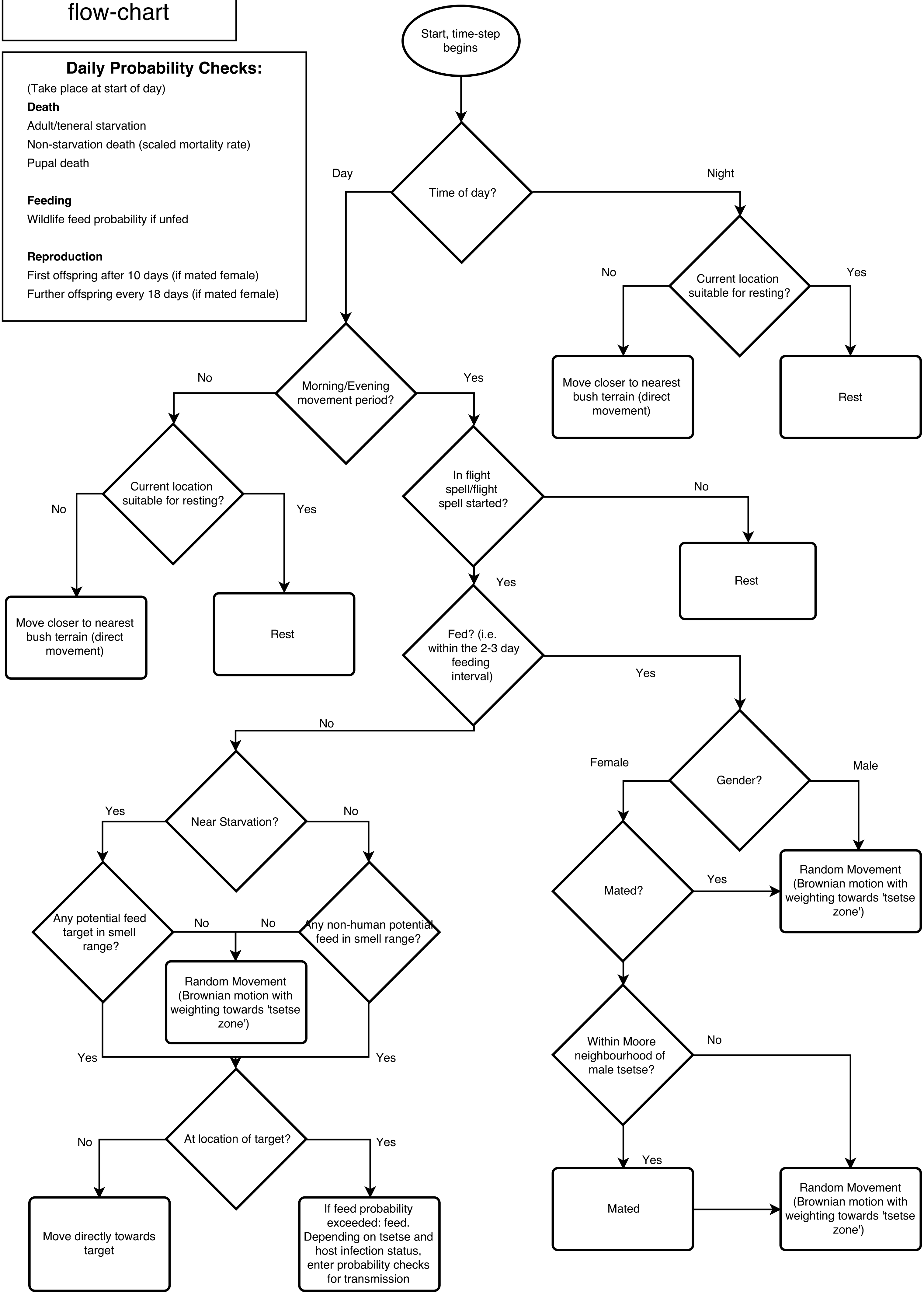

Supplement: S3 File — (PDF) [file pntd.0005252.s005.pdf]
